# The Mediterranean Diet as a Source of Bioactive Molecules with Cannabinomimetic Activity in Prevention and Therapy Strategy

**DOI:** 10.3390/nu14030468

**Published:** 2022-01-21

**Authors:** Riccardo Vago, Francesco Fiorio, Francesco Trevisani, Andrea Salonia, Francesco Montorsi, Arianna Bettiga

**Affiliations:** 1 Division of Experimental Oncology, Urological Research Institute, IRCCS San Raffaele Scientific Institute, 20132 Milano, Italy; fiorio.francesco@hsr.it (F.F.); trevisani.francesco@hsr.it (F.T.); salonia.andrea@hsr.it (A.S.); montorsi.francesco@hsr.it (F.M.); bettiga.arianna@hsr.it (A.B.); 2 Faculty of Medicine and Surgery, Università Vita-Salute San Raffaele, 20132 Milano, Italy

**Keywords:** Mediterranean diet, endocannabinoid system, terpenes, polyphenols, personalized nutrition

## Abstract

The endocannabinoid system is a complex lipid signaling network that has evolved to be a key regulator of pro-homeostatic pathways for the organism. Its involvement in numerous processes has rendered it a very suitable target for pharmacological studies regarding metabolic syndrome, obesity and other lifestyle-related diseases. Cannabinomimetic molecules have been found in a large variety of foods, most of which are normally present in the Mediterranean diet. The majority of these compounds belong to the terpene and polyphenol classes. While it is known that they do not necessarily act directly on the cannabinoid receptors CB1 and CB2, their ability to regulate their expression levels has already been shown in some disease-related models, as well as their ability to modulate the activity of other components of the system. In this review, evidence was gathered to support the idea that phytocannabinoid dietary intake may indeed be a viable strategy for disease prevention and may be helpful in maintaining the health status. In an era where personalized nutrition is becoming more and more a reality, having new therapeutic targets could become an important resource.

## 1. Introduction

The Endocannabinoid System (ECS) is a complex lipid-signaling network that has evolved to be a very important regulator of pro-homeostatic pathways for our organism. Its involvement in pathways such as glucose metabolism, insulin sensitivity and inflammation in the adipose tissue has rendered it a very suitable target for pharmacological studies regarding metabolic syndrome, obesity and other lifestyle-related diseases [1]. ECS activity is based on the interaction between the endocannabinoid molecules N-arachidonoylethanolamine (Anandamide, AEA) and 2-arachidonoylglycerol (2-AG), which are Arachidonic Acid (AA) derivates, and the well-known G-protein coupled cannabinoid receptors 1 (CB1) and 2 (CB2) [2]. CB1 is primarily expressed in the central nervous system and it was thought to be present exclusively in this district before the discovery of functionally relevant expression levels in peripheral organs such as liver, adipose tissue and kidney, among others [3]. CB2 was thought to be absent from the CNS and only present in peripheral organs and in the immune system, but early discoveries were able to pinpoint its presence also in the microglia where it apparently participates in the regulation of neuroinflammation [4,5]. The existence of Δ^9^-tetrahydrocannabinol (THC) responsive receptors had to mean that these receptors had endogenous ligands that would be able to bind them, which led to the discovery of AEA and 2-AG. These endocannabinoid molecules bind with high affinity to both CB1 and CB2 [1], but are also able to bind other non-cannabinoid receptors such as the transient receptor potential vanilloid 1 (TRPV1), peroxisome proliferator-activated receptors (PPARs) and the orphan GPR55, which are all candidates for a possible novel CB3 receptor but still require in-depth studies and CONVINCING results [4,6]. 2-AG is synthesized from membrane phospholipids by phospholipase Cβ and diacylglycerol lipase (DAGL) while its degradation is more complex and consists in the use of at least eight different enzymes, among which we can find monoacylglycerol lipase (MAGL) as the most represented one [7]. AEA’s synthesis relies majorly on the activity of the N-acyl-phosphatidylethanolamine-specific phospholipase D (NAPE-PDL)-like enzyme, while fatty acid amide hydrolase (FAAH) is primarily in charge of its degradation [8].

Given that the ECS is involved in many lifestyle and non-lifestyle related diseases, research on cannabinoids and the medical use of cannabis have become a reality in the scientific community, to the point that some agonists and antagonists of CBRs have already been developed with the idea to mimic the endogenous ligands and induce a modulation of the system in favor of more adequate endocannabinoid expression levels. An example of these is the selective CB1 antagonist Rimonabant, which was able to induce an efficient weight loss in obese patients by lowering ECS tone through CB1 blockade [9]. This drug, however, was removed from the market only one year after its release for unprecedented safety incongruities, such as the high incidence of decreased compliance to the treatment due to depression and suicidal tendencies [10]. Hence, when dealing with the modulation of such an intricate and still misunderstood system, scrupulous evaluation of possible side effects must be accounted for. Novel therapeutic strategies have therefore shifted towards the study of other active principles that could be Rimonabant analogs but may not show its deleterious effects on the CNS. Among the various active principles discovered to have an endocannabinoid effect, we can distinguish distinct classes of molecules: terpenes, flavonoids and polyphenols. These molecules are largely present in the plant kingdom and the Mediterranean diet is particularly rich in some foods that contain them. Among these, Extra-Virgin Olive Oil (EVOO), which is a key element of the Mediterranean diet, is rich in monounsaturated fatty acid (oleic acid in particular) and polyphenolic compounds. In this review, the link between all these classes of compounds and the ECS will be discussed, taking into account the state of the art that is present on the subject. As EVOO is so present in the Mediterranean diet, it is important to define whether the consumption of its bioactive molecules could impact health and quality of life in general and if their relationship with the ECS could contribute to maintaining the health status.

## 2. Dietary Active principles with ECS-Mediated Effects

### 2.1. Terpenes

**Figure 1:**
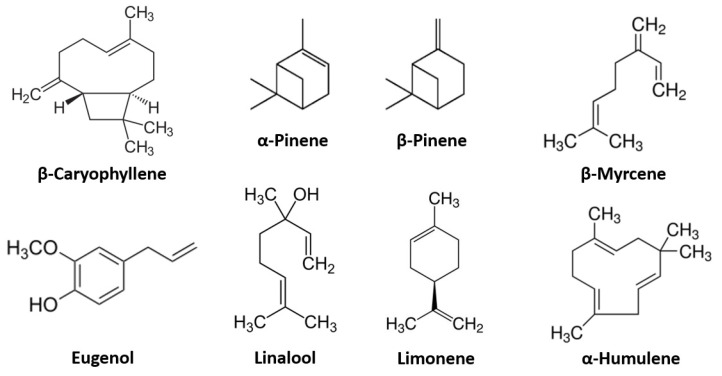
Most represented terpene molecules in literature.

The large interest in the ECS has led to the search for other molecules that can act on its main modulators in order to achieve therapeutic action and a basis for further pharmacological studies. Among all the molecules that have been studied, the ones that attracted the most attention are the terpene compounds, a term also including terpenoids that are oxygenated forms of terpenes [11]. In Cannabis sativa essential oil alone, more than 400 of these molecules have been discovered but only a few of them have been studied to the extent that can create a basic understanding of their actual role in therapeutics [12]. Terpenes are organic volatile compounds that, in the Cannabis plant, are synthesized alongside phytocannabinoids and accumulated in a resin in glandular trichomes, most of which is found on female inflorescences [13]. Their functions in the plant are fundamental for insect and herbivore repellence and pollinator attractance, and their presence is responsible for the plant’s distinctive smell and flavor [14]. Their chemical structure is characterized by pairs of isoprene molecules and the amount of isoprene pairs determines if the terpene is mono, sesqui, di, etc., according to the number of carbons present in the molecule (10, 15 or 20, respectively) [15]. Terpene synthases, which have the ability to synthesize one or multiple terpenes, are the enzymes responsible for the synthesis of these molecules [16]. The simultaneous presence of one or multiple of these enzymes explains the difference in organoleptic properties observed in various varieties of Cannabis sativa. The components of this large class of molecules are presently used in many industrial sectors, including their use as flavors and fragrances in perfumery and cosmetics [17], but are also widely used in medicine for their emerging antioxidant, anti-inflammatory and analgesic properties. Recent research on the use of Cannabis in the medical field has highlighted the possible presence of an entourage effect between terpenes and cannabinoids, which could explain the observation that the administration of whole plant extracts is much more effective than the administration of the single cannabinoid [18]. Given that these molecules are highly represented in several spice and vegetable-derived ingredients, the nutritional interest in these molecules could be quite high. In this section of the review, we will analyze the most known terpenes (structures of the molecules are highlighted in Figure 1) and their relationship with the ECS while also elucidating their presence in different spices commonly used in the Mediterranean area.

#### 2.1.1. β-Caryophyllene

β-Caryophyllene (BCP) is the most represented and promising terpene in the medical and therapeutical research regarding the ECS. BCP is a dietary sesquiterpene found in the essential oil of a large variety of food and spice plants, including cinnamon (*Cinnamon* spp.), oregano (*Oreganum vulgare*), clove (*Eugenia caryphyllata*), thyme (*Salvia* spp.), rosemary (*Rosmarinus officinalis*), hops (*Humulus lupulus*), chili peppers (*Capsicum* spp.) and black pepper (*Piper nigrum*) [19], in addition to up to 35% of *Cannabis sativa* essential oil [20], and has been approved by the FDA as a “generally recognized as safe” dietary additive. The reason for the incredible attention this molecule is attracting is its ability to bind to the Δ^9^-THC binding site of CB2 as a selective agonist in the nanomolar range, while presenting no significant activity towards the CB1 receptor [21]. Numerous pieces of evidence have been gathered over the years regarding the effects that this molecule has as a CB2 agonist and how these effects can be exploited as a therapeutical approach to various diseases. Gertsch et al. [21] in 2008 showed that this ligand–receptor interaction is not only occurring, but it is also active and triggers several pathways, such as cAMP inhibition, MAPK activation, Erk1/2 and JNK1/2 suppression, while also acting as an inhibitor of LPS-stimulated TNF and IL-1β expression in an in vitro experimental model. This CB2 activation, therefore, could imply that BCP modulates inflammatory and other pathophysiological processes by acting directly on the ECS.

BCP was discovered to have efficient analgesic effects in mice models of inflammatory and neuropathic pain [22]. In this study, the effects of oral BCP administration were shown to be CB2 dependent following CB2 blockage by the antagonist SR 144528 and were demonstrated to be effectively higher than the ones obtained when using the known CB2 agonist JWH-133. In fact, the SR 144528 doses required to cancel BCP’s action were markedly higher than the ones used for JWH-133 and, interestingly, BCP’s effects did not induce tolerance but instead appear to strengthen over time. In the same study, BCP was demonstrated to also be effective on carrageenan-induced edema formation, showing CB2-dependent anti-inflammatory effects, and also against thermal hyperalgesia and mechanical allodynia. A very recent study has confirmed the role of BCP as a protective agent against mechanical allodynia in an antiretroviral drug-induced neuropathic pain mice model [23]. The authors suggested that the mechanism through which this effect is achieved is through the inhibition of pro-inflammatory cytokine expression and phospho-ERK1/2 (both of which are elevated in models of neuropathic pain), confirming in an in vivo model the mechanisms earlier proposed by Gertsch et al. The effects of BCP were confirmed by using a CB2, but not CB1, specific antagonist. Since peripheral CB2s receptors are known to have an important role in delaying and acting upon neuropathic pain development [24,25], oral BCP administration could prove to be a useful tool against these types of pain both pharmaceutically and in a tweaked dietary regimen.

CB2 was thought to only be present in the peripheral nervous system for a long time but, since the discovery of this receptor’s presence in the central nervous system [26], there has been a large interest in its role in the modulation of mood disorders, eventually discovering its involvement in anxiety and depression [27]. As a CB2 agonist, BCP was obviously investigated as a novel therapeutic treatment and was found to be effective in reducing anxiety through mechanism non-GABA_A_ and non-5-HT_1A_ receptors-dependent [28]. Interestingly, when BCP was administered in combination with flumazenil (GABA_A_ antagonist) and NAN-190 (5-HT_1A_ antagonist) its anxiolytic effects were maintained. Given this discovery, Bahi et al. [29] set out to elucidate the role of CB2 in this BCP-mediated anxiolytic and antidepressant effects. Once again, they were able to prove CB2 involvement by administration of a CB2 selective antagonist (AM630). The administration of the antagonist showed BCP’s anxiolytic and antidepressant potential in an in vivo model in which mice were challenged through an array of behavioral experiments, such as the elevated plus maze, the open field test and the marble burying test, among others. The antagonist’s ability to reverse BCP’s effects was enough to determine its CB2-mediated efficacy. More recently, a study on BCP-induced alleviation of neurobehavioral changes following a high-fat, high-fructose diet (HFFD) in rats further elucidated the protective role of this molecule [30]. Among other detrimental effects, an HFFD is associated with defective insulin signaling, which causes diet-induced insulin resistance, which in turn is responsible for mood disorders, as well as memory and cognitive deficits. In this study, BCP was able not only to ameliorate fasting blood glucose, insulin resistance and insulin level parameters, but was also able to improve memory and alleviate anxiety and depression in a CB2-dependent manner (proven through AM630 CB2 antagonist administration). The proposed mechanism is a CB2-dependent modulation of the peroxisome proliferator-activated receptor-gamma coactivator 1α (PGC-1α)/Brain-Derived Neurotrophic Factor (BDNF) pathway upregulation. BNDF is a neurotrophin essential for neuronal repair, survival and plasticity, which was previously shown to be downregulated during neurodegenerative diseases and in many inflammatory conditions [31] as a result of oxidative stress and/or TNFα activation [32,33]. The upregulation of this pathway would have a positive effect on the CNS mediated by the ECS. An involvement of BCP-mediated activation of CB2 in the amelioration of central functions has therefore been proven in vivo in animal models, although to which extent these results are translatable to human models is still debated.

BCP also has very interesting effects on the amelioration of the lipid profile by acting on fat deposition and oxidation. In 2013, Zheng et al. [34] showed the involvement of BCP in the CB2-mediated activation of the PGC-1α/SIRT1 pathway and how this activation leads to fatty acid oxidation in vitro. In the same year, Harb et al. [19] showed that 30 mg/kg BCP administration to hypercholesterolemic rats caused an amelioration in the lipid profile with lower serum total cholesterol and LDL and higher HDL with no detectable difference for triglyceride levels. Youssef et al., in their previously cited paper [30], were not only able to prove mood disorder amelioration in HFFD fed rats, but showed that CB2 activation by BCP was also responsible for metabolic changes more positive than the ones relative to the carbohydrate metabolism. BCP treatment was demonstrated to be effective in decreasing visceral fat (in addition to subcutaneous fat, body weight and inflammation which were previously described in the literature [35,36]), in modulating the TNF/NF-κB inflammatory pathway and in upregulating PGC-1α, thereby decreasing oxidative stress markers. PGC-1α, which has been earlier mentioned, has been proven to be of great importance in the crosstalk between CB2 and a protagonist of inflammation and lipid metabolism, Peroxisome Proliferator-Activated Receptor Gamma (PPARγ). In addition to its role in the maintenance of a correct glucose level, PGC-1α acts on PPARγ as a coactivator and is therefore involved in the control of lipid levels and in the synthesis of antioxidant enzymes [37]. BCP’s ability to modulate all of these pathways has been attributed to its agonistic effect on CB2, which in turn activates PGC-1α/SIRT1 and AMPK/CREBl [38]. According to Youssef et al., CB2-mediated upregulation of PGC-1α could represent an interesting mechanism by which BCP can activate PPARγ and all of its effects. The activity of BCP on PPARγ has been debated since it was initially believed not to have a direct action [39], but several recent data indicated a functional relationship. For instance, Galaj et al. [40], by studying cocaine abuse, proved in mice knockout models that PPARα and γ blockade was able to reverse the BCP-induced reduction in cocaine self-administration while CB2 blockade did not, indicating a direct effects of BCP on PPARs. In addition, BCP exposition was able to increase PPARγ expression in an in vitro human chondrocytes model eliciting anti-arthritic effects and this effect was CB2 dependent, since reversed when the CB2 antagonist AM630 was administered [41]. Similarly, the same effect was observed on PPARγ in U373 glioblastoma cell line following BCP administration, which was once again reversed by AM630 [42]. Given the large role PPARs have in many lifestyle-related diseases, such as obesity and diabetes, further studies of the CB2-PPARγ crosstalk could place serious groundwork for a better understanding of the impact that the ECS could have on these diseases and their complications.

#### 2.1.2. The Terpene “Entourage Effect”

Even though BCP has gained a lot of attention, other types of terpenes are emerging as ECS mediators. Among the most studied terpenes, we can find α-pinene (parsley, rosemary, basil and dill), β-pinene (cumin), linalool (lavender, coriander, bay leaves), Eugenol (clove, cinnamon, oregano, ginger, thyme and pepper), β-myrcene (sweet basil, bay leaves, lemongrass, thyme, parsley), α-humulene (sage, ginseng and myrtaceae) and limonene (citrus oils such as orange and tangerine, celery) [13,43,44]. While all of these and many other terpenes have very interesting properties [13,43,45], the scope of this review is to evaluate them on the basis of their interaction with ECS and its mediators, so only those most represented in literature will be discussed. The “entourage effect”, as previously stated, is the hypothesized synergistic effect that terpenes may have with cannabinoids (“inter-entourage” [46]) following the observation that whole-plant extracts are more effective than single cannabinoid administration. This hypothesized effect has led to the belief that these molecules have a regulatory role on the ECS but proving which mechanisms they activate remains the main issue.

Given the lack of studies, Santiago et al. [47] and Finlay et al. [48] set out to prove whether these compounds act as cannabinoid modulators by acting on CBRs in vitro. Santiago et al. tested the effects of six terpenes (α- and β-pinene, β-caryophyllene, β-myrcene, linalool and limonene) on AtT20 cells stably transfected with human CB1 and CB2 and proved that these terpene molecules did not activate CB1 and CB2 when administered alone, nor interfered with Δ-THC’s activity on these receptors. These terpenes also did not modulate the potassium channel’s response caused by the synthetic cannabinoid CP55,940. To our and the same author’s surprise, also BCP did not elicit a CBR-mediated response. The reasons for this are said to be unclear, but they could be justified by the administration of a low dose or by an unproductive coupling between hCB2 and endogenous AtT20 potassium channels, which would require a higher ligand affinity to the receptor. The authors also exclude a possible terpene modulation of Δ[9]-THC’s response by allosteric coupling with CB1, since the negative allosteric modulators used in the study were successful in inhibiting CP55,940 induced hyperpolarization while the terpenes tested were not. The authors are very thorough in their limitations list and propose that future research should focus on multiple pathways and not only the one involving potassium channel activity. Soon after, Finlay et al. [48] published another paper examining the possible terpene–cannabinoid entourage effect on CBRs in vitro, evaluating the activity of the same molecules except for linalool on hCBRs stably transfected HEK cells through a radioligand assay and cAMP signaling assay. The results obtained are consistent with the ones published by Santiago et al., proving no modulation of the response following terpene exposure. Similarly, also in this study, BCP did not show any CB2-dependent activity, although a weak binding to the receptor was demonstrated. Once again, the authors are not sure what the reasons for this result may be but acknowledge the consistency with the other article. These two studies together may present preliminary data showing how terpene direct binding with CBRs is not the mechanism by which a possible entourage effect is mediated, but mechanisms that act on other components of the ECS such as the TRP-channels may be possible.

Following this reasoning, the same research group for the Santiago et al. paper produced another study in which they evaluated the activity of seven terpenoids (BCP, humulene, linalool, α-pinene, β-pinene, β-myrcene and limonene) on human TRPA1 and TRPV1 channel receptors stably transfected in a HEK293 in vitro model [49]. This investigation was conducted on the basis that TRPA1 and TRPV1 have known phytocannabinoid agonists such as Δ^9^-THC and CBD [50] and the endocannabinoids AEA and 2-AG are agonists for both channels [51,52]. The main finding of this study is that none of the seven terpenoids were able to induce a change in internal calcium concentration in either hTRPA1 or hTRPV1 expressing cells. Validation with known agonists proved the reliability of the results since Δ^9^-THC and 2-AG were able to activate TRPA1 expressing cells, while AEA and capsaicin were able to activate the ones expressing TRPV1. Interestingly, while CBD is known to be a TRPV1 agonist [53], this study failed to show its TRPV1 activation ability, and this is explained by the authors as a discrepancy occurring as a result of TRPV1 expression levels or as a result of a difference in testing conditions compared to other studies. Similarly, β-myrcene has also been shown to influence calcium influx by binding to TRPV1 [54] and linalool was able to activate TRPA1 at very high concentrations [55], evidence that does not agree with the findings of this paper. The gray areas regarding the complex interactions between terpenoids and TRP channels are still to be discovered and understood. For example, TRPM8, another TRP channel, is a known target for another well-known terpenoid, menthol (peppermint), and the resulting ligand–receptor binding is responsible for cool-induced analgesia of acute, inflammatory and neuropathic pain [56]. This binding was able to balance the nociceptive effects caused by capsaicin on TRPV1 and Acrolein on TRPA1 [57]. Furthermore, menthol binding to human TRPA1 and TRPV1 cannot be excluded since topical application causes epidermal irritation and inhalation may cause exacerbation of asthma. The majority of terpene compounds are still relatively unknown and, while present-day literature excludes the existence of an entourage effect, further research might be able to clear up some uncertainties and bring precious leads for a better understanding of this complex system.

### 2.2. Polyphenols and Flavonoids

**Figure 2:**
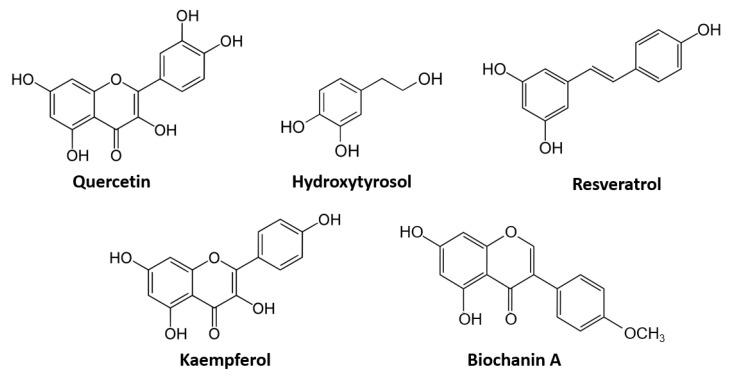
Most represented polyphenol and flavonoid molecules in the literature.

As it can be clear by now, Cannabis Sativa, as well as other plants, contain a plethora of active principles that concur in the modulation of the ECS, and the introduction of these molecules through diet is highly possible. Among these active principles, the class of polyphenols is surely well represented in the literature and contains some of the most promising compounds in nutritional research. Polyphenols are compounds that can be identified by the presence of multiple phenol rings in their structure and this class of molecules is composed of several families and subclasses that also include flavonoids, anthocyanins and stilbenoids [58]. They are largely distributed in plants and their physiological roles include being a fungal and herbivore deterrent and granting UV protection through pigmentation [59,60,61]. Their large presence in a very wide range of plants, including vegetables and fruits and their well-known antioxidant and anti-inflammatory properties make them very interesting molecules that could adapt very well to dietary interventions and personalized nutrition aimed for treatment. Their role as epigenetic regulators for certain genes’ expression has been emerging in the last decade and there is some sparse evidence that links these compounds to the ECS. In this chapter, bibliographic evidence exploring this link will be presented, proving that the involvement of this class of molecules in the modulation of ECS component levels is plausible and that they could take part in a functional and human-applicable intervention strategy for a wide range of diseases. Here, we will examine the literature about the main polyphenols that can be found in the Mediterranean area (structures of the molecules are highlighted in Figure 2) and that could, therefore, be very important in a Mediterranean-oriented dietary profile.

#### 2.2.1. Quercetin

Quercetin [2-(3,4-dihydroxyphenyl)-3,5,7-trihydroxy-4H-chromen-4-one] is present in a large variety of food sources characteristic of the Mediterranean area, such as cherries, apple, red wine, cappers, and red onion [62] and it is the most studied flavonoid. What makes this polyphenolic compound interesting are its numerous beneficial effects [63] and its appreciated versatility in various research fields. It has been discovered to be a potent antioxidant, antibacterial and anti-inflammatory molecule [64,65,66] and it is also believed to have anti-cancer properties [67], despite its poor bioavailability (roughly 2% after a single dose) and complex metabolism [68]. Given all these beneficial effects of this molecule, some authors have started investigating whether this flavonoid is also involved in the modulation of the ECS, specifically the modulation of the CBRs expression and could therefore act on the aforementioned pathways through different routes. While the relationship between Quercetin and CB2 has never been investigated, some evidence is present for CB1. The possibility of a direct ligand–receptor interaction between numerous novel drug candidates and CB1 has been evaluated through homology modeling and structural analysis [69]. Quercetin has demonstrated to have a high affinity towards CB1, comparable to the affinity Rimonabant shows towards the same receptor (−6.56 Kcal/mol with four hydrogen bonds), leading the authors of the study to believe that this specific compound could be a promising and ideal lead molecule for novel obesity treatment, even though only a few studies have explored this relationship at the moment. A paper published in 2015 by Refolo et al. [70] studied the effects of Quercetin on human colon cancer cells (Caco-2 and DLD-I), specifically how this polyphenol is supposed to induce CB1-dependent apoptosis and anti-proliferative effects on tumoral cells. While proving that CB1 expression increased following Quercetin administration, the incomplete inhibition of the effects observed from combined administration of CB1 antagonist SR141716 seems to be a critical point of the study; if CB1 upregulation and Quercetin’s effects on cancer cell proliferation and apoptosis were entirely due to a direct Quercetin–CB1 interaction, the administration of an antagonist should have reverted the effects completely and not only partially. In a follow-up in vivo study conducted by the same research group [71], the same upregulation of CB1 was also observed in mice after dietary treatment with Quercetin while also observing an alteration in expression levels of STAT3 and Bax/Bcl2, suggesting a more protective environment in the intestinal tract of these mice. While criticism could also arise from the fact that all the effects on cancer cell apoptosis and decreased proliferation may be only collateral effects and not entirely CB1-dependent [72], the results of these studies seem to suggest that Quercetin is able to impact the expression of CB1. Since a lower CB1 expression in the intestine seems to be directly correlated with the onset of cancer [73], a dietary treatment with Quercetin aimed at increasing this receptor’s expression in the colon could be a viable therapeutic strategy.

#### 2.2.2. Resveratrol

Resveratrol (3,4,5′-trihydroxy-trans-stillbene) is a polyphenolic compound naturally present in many components of the Mediterranean diet, such as berries, grapes, red wine and other plants [74]. Among its numerous beneficial activities, this molecule is most known for its ability to scavenge free radicals and act on antioxidant pathways [75]. Although it is effectively absorbed in the small intestine, its poor bioavailability due to its fast metabolism in humans has been a big barrier for its possible use as a systemic treatment for numerous pathologies [76,77]. Despite this, resveratrol remains one of the most studied polyphenols in biomedical research to this day. In light of this, this compound has found its place also in ECS-related studies and has produced interesting results. Chen et al. [78] have explored the role of resveratrol in the prevention and attenuation of diet-induced non-alcoholic steatohepatitis (NASH) and found that this polyphenol is able to maintain gut barrier integrity, inhibit gut inflammation and lower LPS-dependent endotoxemia through direct regulation of the ECS. More specifically, they observed that a high-fat diet (HFD) was able to increase CB1 and decrease CB2 mRNA levels in rat distal colon and that resveratrol administration could restore normal expression of the two receptors. Furthermore, they observed that there was a decrease in AEA and an increase in 2-AG levels. This result was significant, since AEA is believed to aggravate the HFD induced gut permeability and inflammation by binding preferentially to CB1, while 2-AG is believed to ameliorate it, since it mainly binds to CB2 in the intestinal tract resulting in anti-inflammatory effects [79,80]. The effects of resveratrol both on CBR and on endocannabinoid molecules appear to be aimed at bringing the respective expressions back to physiological levels, counteracting the detrimental effects that an HFD has on the intestinal environment and, subsequently, on the whole NASH progression.

Beneficial resveratrol-dependent effects on the ECS balance are not limited to the gut environment. Evidence suggests that this polyphenol is also involved in balancing brain levels of endocannabinoids, increasing both AEA and 2-AG when systemically and chronically administered in rats [81]. This evidence is of great importance since raising endocannabinoid levels in the brain has antidepressant and antinociceptive implications and is essential for the neuroprotective actions of resveratrol. Similarly, another study proves that resveratrol can protect the brain from oxidative stress by increasing CB1 and CB2 expression levels in a transient global hypoperfusion rat model [82]. Resveratrol has, therefore, a potential for neuropsychiatric and other cerebral disorders treatment. Following this line of thought, da Costa Oliveira et al. [83] proved that resveratrol has antinociception effects also peripherally in a carrageenan-induced hyperalgesia mouse model and that this effect is CB1-dependent. This dependency was proven by administering CB1 and CB2 antagonists (AM251 and AM630, respectively) and seeing that this administration dose-dependently blocked the resveratrol-induced antinociceptive effect. While AM251 was able to revert the effect, AM630 failed to do so, proving that the antinociceptive effect of resveratrol is CB1 but not CB2 dependent. Resveratrol’s effect in blocking peripheral nociception was enhanced by the administration of MAFP, JZL184 and VDM11 (respectively FAAH, MAGL and endocannabinoid uptake inhibitors), proving once again that increased endocannabinoid levels are effective in the control of both central and peripheral nociception mechanisms. This study also proved that the opioid and the endocannabinoid systems may be connected, and that resveratrol exerts its antinociceptive action through both these systems. While CB1, CB2 and μOR’s expressions were unaltered in this study, the authors explain that it may be due to the fact that Resveratrol was administered acutely and not chronically, and that this type of administration may be unable to act on the receptor’s expression. It may be interesting to evaluate these receptors’ expressions following chronic Resveratrol administration in the same model. Taken together, these results outline Resveratrol’s role as a modulator for the expression of many ECS components and could be used as a drug for numerous other chronic disorders.

#### 2.2.3. Kaempferol and Biochanin A

As we have already seen in this review, the modulation of the expression of CB1 and CB2 is not the only option through which we can alter the balance of the ECS. Interfering with the activity of enzymes that are part of the ECS is, in fact, another possible way to turn the tides. In particular, some polyphenols have shown an inhibiting effect on the FAAH, the enzyme responsible for AEA degradation, prolonging the duration of AEA effects [84]. Chronic stress animal models show a significant decrease in AEA levels in all brain areas [85] and the dysregulation of endocannabinoid levels were associated with numerous psychiatric disorders [86]; therefore, an enhancement in endocannabinoid signaling due to FAAH inhibition could be used as a novel phytotherapeutic treatment for anxiety and stress relief. Polyphenols such as kaempferol and biochanin A have displayed interesting properties. Kaempferol is a natural flavone present in beans, broccoli cabbage, grapes, tomatoes and citrus, among other plant sources, while biochanin A is mostly found in red clover and legumes, such as chickpeas, soy and peanuts. Among other naturally occurring compounds, kaempferol appears to be the most potent FAAH inhibitor [87]. In a recent study by Ahmad et al. [88], this effect was confirmed and, in particular, kaempferol displayed dose-dependent effects on FAAH inhibition in vitro, as well as reduction in the freezing response in contextual fear-conditioned rats. In the same study, the treatment with kaempferol also showed anxiolytic effects comparable to those observed after standard treatment with diazepam, which were reversed by CB1 antagonist administration. These results confirm previous studies which asserted that ECS modulation by either FAAH inhibition and CB1 activation could reduce stress and fear-related behavior in rodents [89,90]. Biochanin A has also shown promising effects as a FAAH inhibitor but, in a paper published by Thors et al. [91], it also inhibited the binding of a CB receptor agonist ([^3^H]CP55,940) to brain CB1 and recombinant CB2 receptors. While the effects of biochanin A on CBRs were modest, a connection between this molecule and the ECS has been presented, paving the way for future research. Even though dietary intake of these two polyphenols is very scarce and would hardly be enough to be pharmacologically active, they remain a valid option for possible nutraceutical treatments or for supplementation in general.

#### 2.2.4. The Synergistic and Matrix Effects of Polyphenols

If the administration of single polyphenols is able to elicit changes in ECS components’ expressions, it is reasonable to believe that more molecules combined would be able to induce more evident changes. Since the bioavailability of these compounds is limited, a synergistic effect of different molecules with virtually the same function could potentially be able to counteract what is lost during physiological metabolic and detoxification mechanisms. We also know that the food matrix in which dietary phytochemicals are present plays an important role in their absorption and availability, and we know this to be true also for polyphenols [87,92]. Following this line of reasoning, Cocci et al. [93] recently published a study in which they evaluated the effect of a tart cherry supplement on visceral fat ECS components’ expression and its impact on adipogenesis-related genes in a rat model for diet-induced obesity (DIO) following a high-fat diet (HFD). They showed that tart cherry is a fruit very rich in polyphenols, especially anthocyanins, quercetin, kaempferol and other flavonoids [94,95] and it is of interest for this review since this fruit is popular in the Mediterranean area. The results they have obtained are very interesting and promising. While having no effect on body weight and adipocyte size, the supplement appeared to be effective in downregulating CB1 (which is upregulated and over-stimulated in the visceral fat of obese individuals [96]) and adipogenic genes such as PPARγ and SREBP-1c, while also upregulating adipocyte differentiation inhibitor PREF-1. The authors explain that the anti-adipogenic effect presented by the tart cherry supplement may be due to its ability to downregulate CB1, which seems to be directly related to the PPARγ pathway. This crosstalk between the ECS and PPARγ could outline, therefore, an interesting potential prophylactic role of phytochemicals in adipogenesis regulation. Some studies, however, have suggested that CB1 may be upregulated in response to the pro-inflammatory environment [97,98] and that, therefore, the subsequent CB1 downregulation after the tart cherry extract may be due to a change in the inflammation that may be caused by the polyphenols’ known anti-inflammatory properties. Further studies are required to tie all these pieces of evidence together, thereby making the pathways and crosstalk clearer in order to build enough plausibility and move towards the human model.

#### 2.2.5. Olive Oil, Hydroxytyrosol and the ECS

The Mediterranean diet is one of the most known diets in the world for its cultural and territorial value and has been recognized as an intangible cultural heritage of humanity by UNESCO in 2010 (https://ich.unesco.org/, accessed on 5 February 2021). Since the publication of Ancel Keys’ Seven Country Study in the 1960s, in which for the first time he was able to prove the strong protective effects this diet has towards cardiovascular events despite its high-fat content, the Mediterranean diet entered the nutritional guidelines worldwide as part of a healthy lifestyle. In 2019, it was recognized by the EAT-Lancet Commission as a possibly universal model of a healthy diet (https://eatforum.org/eat-lancet-commission/, accessed on 5 February 2021). During the second “Ancel Keys” International Seminar [99], which took place in 2021, this diet was discussed in detail and its relevance as an effective and preventive dietary approach is still highly considered in the scientific community. What makes the Mediterranean diet special is its positive impact on several metabolic disorders and cardiovascular risk factors, such as hypercholesterolemia, metabolic syndrome and diabetes, and other important pathologies such as cancer [100,101,102], despite being a diet highly reliant on fat. This diet is rich in monounsaturated fatty acids, which should account for the majority of total daily energy derived from lipids, as opposed to saturated fatty acids, which should be kept <10% of the total daily energy intake. This high energy intake deriving from monounsaturated fatty acids is explained by the consumption of large quantities of olive oil, which over the years has always been described as one of the key components of the Mediterranean diet, along with vegetables and fruits, whole grain cereals, nuts and legumes [103]. Extra-virgin olive oil (EVOO) is naturally rich in polyphenolic compounds, which we recognize from previous sections as a family of molecules known for their antioxidant, anti-inflammatory, neuroprotective and anti-obesity properties [104,105,106]. Among these molecules, we can find Hydroxytyrosol (OHT), which has displayed similar properties [107]. With the rising interest in dietary ECS modulation within the scientific community, olive oil and OHT have also been candidates for research on this topic. While EVOO and OHT are fairly unrepresented in the literature, there is some sparse evidence that links these molecules to the modulation of the main ECS components, mainly the expression of CB1 and CB2.

For what concerns CB1, there are two main papers that focus on the role of OHT and EVOO in the modulation of this receptor. Di Francesco et al. [108] in 2014 demonstrated that EVOO, Oil Phenolic Extract (OPE) and OHT taken singularly were able to upregulate CB1 gene expression both in in vitro and in vivo models by acting through epigenetic mechanisms. In the in vitro studies, EVOO extract, OHT and OPE were administered to human colon cancer cells (Caco-2) and *CNR1* gene expression was evaluated by quantifying both CB1 mRNA and protein. They found a significant upregulation of *CNR1* at 24 h, confirmed by Western-blot analysis; however, no short-term (4 h) effects were noticed and at 48 h, the expression values went back to baseline, suggesting EVOO, OHT and OPE exposure caused a transient upregulation of the gene. To confirm these data, an evaluation of *CNR1* gene methylation was carried out, demonstrating a significant reduction in methylation levels of the *CNR1* promoter caused by EVOO, more specifically by its phenolic components. In fact, treatment with EVOO deprived of the phenolic fraction produced no methylation changes and no CB1 upregulation. CB1 upregulation in this cellular model is particularly interesting since the *CNR1* gene is silenced in colorectal cancer cells compared to normal colon mucosa cells (NCM460) and this gene has already been demonstrated to act as a tumor suppressor in vivo [73]. The trend observed in vitro on Caco-2 cells was confirmed by the analysis of colon samples of rats subjected to 10-day treatment with EVOO, with administrations repeated daily. On the contrary, no significant upregulation in any of the other ECS components tested (CB2, TRPV1, GPR55, MAGL and NAPE-PLD, among others) was noticed. Taken together, these results demonstrate that the phenolic fraction of EVOO is responsible for the epigenetic changes observed for *CNR1* in Caco-2 and in rat colon in a transient manner at 24 h. Furthermore, the observed *CNR1* silencing in colorectal cancer is an interesting starting point for investigation on other cancer types.

Opposite evidence comes from a study published by Tutino et al. [109] in which they proved that OHT down-regulated *CNR1* expression in a 3T3-L1 preadipocyte cell model. The role of CB1 in adipocytes has been proven to be opposite to the one observed in the previous study, since it has been previously seen that CB1 is overexpressed in visceral adipose tissue of obese individuals and can also produce and release endocannabinoids, thereby increasing AEA and 2-AG serum levels [110,111]. The results of this study are, therefore, not contradictory compared with the previous paper, but highlight a possible tissue-specific role of this receptor. The authors were also able to observe a down-regulation in PPARγ, which is involved in preadipocyte differentiation and in lipid storage. Together, these results suggest that OHT administration may be an effective treatment for obesity, helping weight loss either through a direct action on CB1 or through an alternative pathway involving PPARγ that results in CB1 inhibition. A reduction in weight leads to an amelioration of obesity and the resulting constitutive low-grade inflammation, thereby improving the quality of life and exerting an indirect protection against tumors. These two studies outline a clear role of EVOO in the tissue-specific modulation of CB1 expression levels through epigenetic and possibly direct mechanisms.

Proof that CB2 expression can be modulated by EVOO or by its polyphenolic components is lacking in the literature. The previously cited study by Di Francesco et al. [108] also did not find a significant impact of EVOO, OHT and OPE on CB2 expression. The only evidence of EVOO-dependent CB2 expression modulation comes from a single in vivo study, published in 2016 by Notarnicola et al. [112]. Researchers treated C57BL/6J Apc^Min/+^ mice (a model for experimental colorectal carcinogenesis; mice with this mutation are predisposed to the development of multiple intestinal adenomas) with different FA-enriched diets for a period of 10 weeks. One group was administered with an AIN-93M standard diet in which soy oil was replaced with olive oil and all groups were fed an isocaloric diet. Through gene expression and Western-blot analysis the authors were able to conclude that all diets (olive oil, omega-3 and omega-6) exhibited anti-inflammatory effects in the adipose tissue by inducing CB2 and upregulating nitric oxide synthase 1 (NOS1), both participating in the prevention of diet-induced obesity. What makes these results even more interesting is that CB2 expression seems to be NOS1 dependent [113]. Among all the different diets, the EVOO diet was the one that had the greatest effects on CB2 and NOS1 expression. The authors explain how this effect may be due to the high MUFA content, while polyphenols such as OHT were not mentioned. Evaluating the impact these components may have on CB2 expression could represent an interesting future research topic.

## 3. Microbiota and ECS

The involvement of the intestinal microbiota in the progression and onset of several diseases has been one of the most studied topics in the last decade. Many links between the composition of this delicate ecosystem and a number of diseases have already been found and this led researchers to investigate it further. It is now highly recognized that the diet plays a crucial role in the selection of bacterial species in the gut, with different dietary profiles acting differently on the microbiota composition and affecting human health positively or negatively as a consequence [114,115,116]. The Mediterranean diet is no exception to this rule, as it has been demonstrated to positively modulate the gut microbiota composition in humans, primates and rodents [117]. These effects have been linked to dietary fiber, mono- and poly-unsaturated fatty acids and antioxidants, which are highly represented in this dietary profile [118]. Being rich in cannabinomimetic molecules, the next big step in the study of the endocannabidiome was to see if the gut microbiota can be influenced by the modulation of the system possibly elicited by these compounds or if the observed benefits of a Mediterranean diet on this complex ecosystem are independent of them. In the next section, we describe a two-way relationship between the modulation of the ECS and the gut microbiota composition and how by altering one’s activity, we could positively influence the other, leading to positive outcomes on lifestyle-related pathologies.

### 3.1. Modulation of the ECS Alters the Microbiota Composition

Recent studies have proven that targeting the ECS directly can lead to an alteration in the composition of the gut microbiota in favor of species with a positive impact on health. It was seen that the microbiota and the endocannabidiome cooperate in a series of intertwined pathways, which, when disrupted, can worsen preexisting low-grade inflammation and insulin resistance in obese patients [119]. The involvement of CB1 in intestinal and metabolic homeostasis has been studied in detail, identifying its antagonism as a possible way to improve gut barrier function. A higher ECS tone has been associated with an increase in gut permeability and treatment with a CB1 agonist HU-210 induced, as a consequence, severe metabolic disturbances such as glucose intolerance, lipid accumulation in the muscle and endotoxemia [79]. Bahrami et al. [120] have proven for the first time that CB1 blockade improves colonic inflammation, systemic inflammation and insulin resistance in diet-induced obesity (DIO) mice fed with a high-fat diet and treated with Rimonabant (SR141716A), a CB1 antagonist. Interestingly, CB1 antagonist administration also altered the gut microbiota composition in favor of more protective species such as *Akkermansia muciniphila*, which is known to ameliorate DIO and diabetes parameters such as endotoxemia, adiposity, glucose metabolism and insulin resistance when transferred live in mouse models [121]. This species’ abundance was suggested to be restored as a consequence of increased expression of MUC2, a transcription factor in charge of host mucin production regulation. Mucin is the main nutrient source for *A. muciniphila* and is essential for its growth. These outcomes were demonstrated to be rimonabant administration-dependent in obese mice and were also proven to be independent from caloric restriction and weight loss. In addition to increased abundance in *A. muciniphila*, the authors observed a decrease in the *Lachnospiraceae* and *Erysipelotrichaceae* families. This is a significant finding, as these two bacterial families belonging to the *Firmicutes* phylum are thought to be involved in weight gain and metabolic syndrome induction [122], but also in diabetes [123] and inflammation-related GI disorders [124]. What appears to make the link between CB1 antagonism and gut microbiota even stronger is the increased production of butyric and propionic acid evaluated by Bahrami et al. by conducting gas chromatography on the mice’s cecal material. This increased production of short chain fatty acids can be explained by an increased abundance of beneficial butyrogenic and propionogenic species following the administration of Rimonabant. *A. muciniphila* is a prominent example of this statement, as propionic acid is its main metabolite. This interpretation, however, remains a hypothesis, as the authors believe the effects that Rimonabant had on the composition of the gut microbial community *in toto* could be secondary to its effect on the inflammatory state, which then led to a change in the environmental characteristics of the intestine.

If targeting CB1 directly by using synthetic or natural molecules is a viable strategy for tweaking both its activation levels and the gut microbial community composition, it can be hypothesized that targeting other receptors of the endocannabidiome can also have positive effects. N-Acylethanolamines (NAEs) are fatty acid amides that share synthesis and degradation enzymes with endocannabinoid molecules and, even though they do not act on the cannabinoid receptors, they are active on TRPV1, PPARs and GPR119 and GPR55, which have been associated with the same effects as CB2 (improvement of glucose intolerance, perturbed intestinal permeability, insulin resistance and obesity) and are part of the endocannabidiome receptors [125,126]. NAPE-PLD is the most important NAE-synthesizing enzyme and its active presence in the adipose tissue has been proven [127]. Geurts et al. [128] have demonstrated that NAEs synthesized in the adipose tissue by NAPE-PLD interfere with the microbiota, since the knock-out (KO) of adipose tissue-specific NAPE-PLD gene in obese mice greatly shifted the composition of gut microbiota, independently from diet diversity (high-fat diet and control diet). The probiotic Lactobacillus and Allobaculum genera were decreased in control diet-fed KO mice in respect to WT mice fed with the same diet, underlying the crucial role of NAPE-PLD in a healthy microbiota selection. Moreover, when transferring microbiota from NAPE-PLD KO mice to germ-free recipients, the whole phenotype was replicated, thus indicating that the changes in the microbiota have a key role in the phenotype definition. This study demonstrates that NAEs have an impact on the gut microbial community that may or may not be mediated by the previously mentioned non-cannabinoid receptors. By analyzing these articles, we can therefore assume that a selection of a healthier microbiota could be achieved by modulating both CRs and non-CRs ECS inputs. It is still unclear, however, how these changes in ECS levels directly affect the microbiota and which pathways and receptors are activated in order to achieve the observed results. Hence, even if further investigation is required, these studies lay robust groundworks for mechanistic studies aimed at shining more light on the topic.

### 3.2. Alteration of the Microbiota Can Modulate the ECS

If ECS targeting can cause changes in the microbiota, can the modulation of the microbial community positively affect the ECS and its levels of activation? The gut microbiota’s influence on the ECS has been proven to take place on several occasions, with certain species that are able to modulate endocannabinoid plasma levels both positively and negatively. We have previously seen how the introduction of live *A. muciniphila* in mice models has positive effects on DIO and diabetes. What is also interesting about an increased abundance of this microorganism is that the plasma levels of endocannabinoid 2-AG and two of its congeners 2-OG and 2-PG were also increased concomitantly. 2-AG is known to have positive effects on intestinal homeostasis by reducing metabolic endotoxemia, peripheral and brain inflammation and circulating levels of pro-inflammatory cytokines and has been demonstrated to protect against trinitrobenzene sulfonic acid-induced colitis in mice [129]. This evidence suggests, therefore, its role as a “gate keeper”, meaning that it has a protective function against the exacerbation of gut permeability, opposite to the one of AEA, which is thought to be a “gate opener” [121]. While a lot is known about 2-AG, little can be said about the role of the other two species in the maintenance of gut homeostasis. There are authors that suggest a link between 2-OG and the orphan GPR119 [126], and others that suggest that these 2-AG congeners may serve as functional CB1 antagonists [130]. Either way, the observed increase in these molecules proves a close relationship between *A. muciniphila* and the ECS and lays an important foundation for future therapeutic strategies.

In the previously discussed paper, Bahrami et al. showed an increase in butyrate and propionate following the administration of Rimonabant and suggested it to be a consequence of increased butyrogenic and propionogenic microorganism abundance. Other authors set out to prove the opposite relationship, specifically if an increased butyrate and propionate production could affect ECS-related expression levels. In 2017, Kang et al. [131] tested the effect of dietary capsaicin on the gut microbiota of high-fat diet-fed obese mice. A beneficial effect of this molecule was observed on the mice microbiota composition, changing its profile in favor of butyrogenic species such as Clostridium clusters IV (*Ruminococcaceae*) and XIV (*Lachnospiraceae*, including *Roseburia* spp.) and against LPS-synthesizing Gram-negatives. Butyric acid is known to be beneficial for gut health, diminishing low-grade inflammation and positively modulating gut permeability. Lower gut permeability hampers LPS translocation in plasma, thereby lowering endotoxemia and diminishing adipose tissue endocannabinoid production. As a consequence of this ECS tone cut, CB1 is downregulated, feeding into a cycle of positivity for gut health. Capsaicin seems to have protective properties in the high-fat diet-fed obese mice, since the administration of this molecule in association with HU-210 (CB1 agonist) mitigated the disruptive effects of the single administration of the agonist. To prove that these effects were associated with microbiota composition, fecal transplantation was carried out on germ-free mice and, following the procedure, the anti-metabolic endotoxemia effects were replicated. As for capsaicin, there are other compounds able to positively modulate the gut microbiota towards a more butyrsogenic profile, among which we can find multiple prebiotic fibers [132,133,134]. If further investigations are able to confirm this trend, the enhancement of these species could also be a potential strategy for ECS tone correction in obesity.

Whether one or multiple species are responsible for the impact on the ECS is still a very debated topic but, as time proceeds, numerous links are being discovered. A recent article by Markey et al. [135] explored the impact of *Candida albicans* on the gut-brain axis and its ability to dysregulate the balance of the ECS. It has been seen that *C. albicans* colonization, while protecting the gut’s health against pathobionts, induces an AEA-CB1 deficit which increases both stress-induced and basal corticosterone production related to anxiety-like behavior. By administering a FAAH blocker (URB597) to *C. albicans* colonized mice, the trend was reversed, while no effect was noted in mock-colonized mice. K-means cluster analysis supported the hypothesis that the AEA deficit was responsible for the changes in behavior, which was further proven by the increased abundance of two other NAEs (linoleoyl and linoleoyl ethanolamine) in the cecum of *C. albicans* colonized mice. The authors explain that the change in precursor abundance in the GI tract noticed through feeding studies could contribute to the alterations in AEA levels that were observed in this study. Despite not being involved in the lifestyle-related diseases that are in study in this review, this is an example of how there could be undiscovered links between certain species of the microbial community and a healthier ECS equilibrium. While Markey et al. showed that *Candida albicans* alone seems to have the ability to modulate the ECS, Lacroix et al. [136] showed that there is a strong time-dependent association between the abundance of several bacterial genera of the intestinal microbiota and the concentration of AEA and 2-AG in the ileum and plasma of high-fat high sugar (HFHS) diet-fed mice. This study also showed a decrease in CB2 expression in the early stages of the HFHS diet, which could have shifted the ECS mediator profile to preferential binding to CB1, which then increased intestinal permeability, inflammation, insulin resistance and may have led to a subsequent change in the composition of the microbiota. It is undeniable that there are numerous undiscovered details that need to be clarified by future studies, but these observations confirm that there is some kind of cooperation between single microorganism species that, each with its own metabolism, can contribute to a healthy gut environment by acting on the ECS.

## 4. Conclusions

The ECS has been rightfully gaining attention as a pharmaceutical target for numerous diseases. Literature links this complex lipid signaling system to a diverse array of conditions affecting human health and the modulation of said system has been proven to be possible. Nature provides us with a large number of molecules that are either able to bind these receptors directly or modulate their expression by acting on other ECS components. We have discussed the most well-known molecules that have demonstrated to possess some affinity for the system. Interestingly, many other phytocannabinoids share structures with endocannabinoids and theoretically could be active in eliciting an ECS-mediated response, but the absorption and metabolism add a degree of complexity that has to be taken into account when talking about dietary intervention. The goal of a specific dietary regimen or a functional food is far from a pharmaceutical treatment, but this does not exclude the possibility of a combined approach with a therapeutic regime from a prevention standpoint. By taking some edge off the collateral effects that some therapeutical approaches inevitably lead to and by potentially raising compliance, a combined approach involving phytocannabinoids may be an option in the future. What also makes research on the ECS challenging is the fact that the role of its activation in every tissue has not yet been fully understood: while there are some diseases such as obesity and dyslipidemia that may benefit from a reduction in the ECS tone, neuropsychiatric diseases and mood disorders may arise from a reduction in ECS activation in certain brain areas such as the hypothalamus or the hippocampus, and the Rimonabant case has taught us to be weary of this double-faced coin. Despite this, ECS research seems promising all around and, in the nephrological field, CB2 activation has been proven to potentially prevent or ameliorate diabetic nephropathy [137,138]. For these reasons, the presence of cannabinomimetic molecules in the diet cannot be overlooked and new discoveries are needed to shine a light onto this mysterious system, to better comprehend its role and its potential. In an era where personalized nutrition is becoming more and more a reality, having new therapeutic targets could become a large resource and would help us to define new strategies to tackle unresolved issues.

## Data Availability

Not applicable.

## References

[1] Di Marzo V., Silvestri C. (2019). Lifestyle and Metabolic Syndrome: Contribution of the Endocannabinoidome. Nutrients.

[2] Gertsch J. (2017). Cannabimimetic phytochemicals in the diet—An evolutionary link to food selection and metabolic stress adaptation?. Br. J. Pharmacol..

[3] Tam J., Hinden L., Drori A., Udi S., Azar S., Baraghithy S. (2018). The therapeutic potential of targeting the peripheral endocannabinoid/CB 1 receptor system. Eur. J. Intern. Med..

[4] Pacher P., Mechoulam R. (2011). Is lipid signaling through cannabinoid 2 receptors part of a protective system?. Prog. Lipid Res..

[5] Atwood B., Mackie K. (2010). CB2: A cannabinoid receptor with an identity crisis. J. Cereb. Blood Flow Metab..

[6] Tsuboi K., Uyama T., Okamoto Y., Ueda N. (2018). Endocannabinoids and related N-acylethanolamines: Biological activities and metabolism. Inflamm. Regen..

[7] Murataeva N., Straiker A., Mackie K. (2014). Parsing the players: 2-arachidonoylglycerol synthesis and degradation in the CNS. J. Cereb. Blood Flow Metab..

[8] Maccarrone M. (2017). Metabolism of the Endocannabinoid Anandamide: Open Questions after 25 Years. Front. Mol. Neurosci..

[9] Samat A., Tomlinson B., Taheri S., Thomas G.N. (2008). Rimonabant for the Treatment of Obesity. Recent Pat. Cardiovasc. Drug Discov..

[10] Soyka M. (2008). Rimonabant and Depression. Pharmacopsychiatry.

[11] Ferber S.G., Namdar D., Hen-Shoval D., Eger G., Koltai H., Shoval G., Shbiro L., Weller A. (2020). The “Entourage Effect”: Terpenes Coupled with Cannabinoids for the Treatment of Mood Disorders and Anxiety Disorders. Curr. Neuropharmacol..

[12] Gershenzon J., Dudareva N. (2007). The function of terpene natural products in the natural world. Nat. Chem. Biol..

[13] Nuutinen T. (2018). Medicinal properties of terpenes found in Cannabis sativa and Humulus lupulus. Eur. J. Med. Chem..

[14] Russo E.B., Marcu J., Kendall D., Alexander S.P.H. (2017). Cannabis Pharmacology: The Usual Suspects and a Few Promising Leads. Advances in Pharmacology.

[15] Wagner H. (2011). Synergy research: Approaching a new generation of phytopharmaceuticals. Fitoterapia.

[16] Booth J.K., Yuen M.M.S., Jancsik S., Madilao L.L., Page J.E., Bohlmann J. (2020). Terpene Synthases and Terpene Variation in Cannabis sativa. Plant Physiol..

[17] Singh B., Sharma R.A. (2014). Plant terpenes: Defense responses, phylogenetic analysis, regulation and clinical applications. 3 Biotech.

[18] Russo E.B. (2019). The Case for the Entourage Effect and Conventional Breeding of Clinical Cannabis: No “Strain,” No Gain. Front. Plant Sci..

[19] Harb A.A., Bustanji Y., Abdalla S.S. (2018). Hypocholesterolemic effect of β-caryophyllene in rats fed cholesterol and fat enriched diet. J. Clin. Biochem. Nutr..

[20] Malingre T., Hendriks H., Batterman S., Bos R., Visser J. (1975). The essential oil ofcannabis sativa. Planta Med..

[21] Gertsch J., Leonti M., Raduner S., Racz I., Chen J.-Z., Xie X.-Q., Altmann K.-H., Karsak M., Zimmer A. (2008). Beta-caryophyllene is a dietary cannabinoid. Proc. Natl. Acad. Sci. USA.

[22] Klauke A.-L., Racz I., Pradier B., Markert A., Zimmer A., Gertsch J. (2014). The cannabinoid CB2 receptor-selective phytocannabinoid beta-caryophyllene exerts analgesic effects in mouse models of inflammatory and neuropathic pain. Eur. Neuropsychopharmacol..

[23] Aly E., Khajah M.A., Masocha W. (2020). β-Caryophyllene, a CB2-Receptor-Selective Phytocannabinoid, Suppresses Mechanical Allodynia in a Mouse Model of Antiretroviral-Induced Neuropathic Pain. Molecules.

[24] Beltramo M. (2009). Cannabinoid Type 2 Receptor as a Target for Chronic-Pain. Mini-Rev. Med. Chem..

[25] Guindon J., Hohmann A.G. (2008). Cannabinoid CB2receptors: A therapeutic target for the treatment of inflammatory and neuropathic pain. Br. J. Pharmacol..

[26] Van Sickle M.D., Duncan M., Kingsley P.J., Mouihate A., Urbani P., Mackie K., Stella N., Makriyannis A., Piomelli D., Davison J.S. (2005). Identification and Functional Characterization of Brainstem Cannabinoid CB 2 Receptors. Science.

[27] Marco E.M., García-Gutiérrez M.S., Bermúdez-Silva F.-J., Moreira F., Guimarães F., Manzanares J., Viveros M.-P. (2011). Endocannabinoid system and psychiatry: In search of a neurobiological basis for detrimental and potential therapeutic effects. Front. Behav. Neurosci..

[28] Galdino P.M., Nascimento M.V.M., Florentino I.F., Lino R.C., Fajemiroye J.O., Chaibub B.A., de Paula J.R., de Lima T.C.M., Costa E.A. (2012). The anxiolytic-like effect of an essential oil derived from Spiranthera odoratissima A. St. Hil. leaves and its major component, β-caryophyllene, in male mice. Prog. Neuro-Psychopharmacol. Biol. Psychiatry.

[29] Bahi A., Al Mansouri S., Al Memari E., Al Ameri M., Nurulain S.M., Ojha S. (2014). β-Caryophyllene, a CB2 receptor agonist produces multiple behavioral changes relevant to anxiety and depression in mice. Physiol. Behav..

[30] Youssef D.A., El-Fayoumi H.M., Mahmoud M.F. (2018). Beta-caryophyllene alleviates diet-induced neurobehavioral changes in rats: The role of CB2 and PPAR-γ receptors. Biomed. Pharmacother..

[31] Eyileten C., Kaplon-Cieslicka A., Mirowska-Guzel D., Małek L., Postula M. (2017). Antidiabetic Effect of Brain-Derived Neurotrophic Factor and Its Association with Inflammation in Type 2 Diabetes Mellitus. J. Diabetes Res..

[32] Jung J.I., Kim E.J., Kwon G.T., Jung Y.J., Park T., Kim Y., Yu R., Choi M.-S., Chun H.S., Kwon S.-H. (2015). β-Caryophyllene potently inhibits solid tumor growth and lymph node metastasis of B16F10 melanoma cells in high-fat diet–induced obese C57BL/6N mice. Carcinogenesis.

[33] Xu H., Czerwinski P., Xia N., Förstermann U., Li H. (2015). Downregulation of BDNF Expression by PKC and by TNF-? in Human Endothelial Cells. Pharmacology.

[34] Zheng X., Sun T., Wang X. (2013). Activation of type 2 cannabinoid receptors (CB2R) promotes fatty acid oxidation through the SIRT1/PGC-1α pathway. Biochem. Biophys. Res. Commun..

[35] Kishi T., Hirooka Y., Nagayama T., Isegawa K., Katsuki M., Takesue K., Sunagawa K. (2015). Calorie Restriction Improves Cognitive Decline via Up-Regulation of Brain-Derived Neurotrophic Factor. Int. Heart J..

[36] Rossi F., Bellini G., Luongo L., Manzo I., Tolone S., Tortora C., Bernardo M.E., Grandone A., Conforti A., Docimo L. (2016). Cannabinoid Receptor 2 as Antiobesity Target: Inflammation, Fat Storage, and Browning Modulation. J. Clin. Endocrinol. Metab..

[37] Besseiche A., Riveline J.-P., Gautier J.-F., Bréant B., Blondeau B. (2015). Metabolic roles of PGC-1α and its implications for type 2 diabetes. Diabetes Metab..

[38] Sharma C.M., Al Kaabi J.M., Nurulain S.N., Goyal S., Amjad Kamal M., Ojha S. (2016). Polypharmacological Properties and Therapeutic Potential of β-Caryophyllene: A Dietary Phytocannabinoid of Pharmaceutical Promise. Curr. Pharm. Des..

[39] Mueller M., Jungbauer A. (2009). Culinary plants, herbs and spices—A rich source of PPARγ ligands. Food Chem..

[40] Galaj E., Bi G.-H., Moore A., Chen K., He Y., Gardner E., Xi Z.-X. (2020). Beta-caryophyllene inhibits cocaine addiction-related behavior by activation of PPARα and PPARγ: Repurposing a FDA-approved food additive for cocaine use disorder. Neuropsychopharmacology.

[41] Irrera N., D’Ascola A., Pallio G., Bitto A., Mazzon E., Mannino F., Squadrito V., Arcoraci V., Minutoli L., Campo G.M. (2019). β-Caryophyllene Mitigates Collagen Antibody Induced Arthritis (CAIA) in Mice Through a Cross-Talk between CB2 and PPAR-γ Receptors. Biomolecules.

[42] Irrera N., D’Ascola A., Pallio G., Bitto A., Mannino F., Arcoraci V., Rottura M., Ieni A., Minutoli L., Metro D. (2020). β-Caryophyllene Inhibits Cell Proliferation through a Direct Modulation of CB2 Receptors in Glioblastoma Cells. Cancers.

[43] Baron E.P. (2018). Medicinal Properties of Cannabinoids, Terpenes, and Flavonoids in Cannabis, and Benefits in Migraine, Headache, and Pain: An Update on Current Evidence and Cannabis Science. Headache J. Head Face Pain.

[44] Khalil A.A., Rahman U.U., Khan M.R., Sahar A., Mehmood T., Khan M. (2017). Essential oil eugenol: Sources, extraction techniques and nutraceutical perspectives. RSC Adv..

[45] Gonçalves E.C.D., Baldasso G.M., Bicca M.A., Paes R.S., Capasso R., Dutra R.C. (2020). Terpenoids, Cannabimimetic Ligands, beyond the Cannabis Plant. Molecules.

[46] Nallathambi R., Mazuz M., Namdar D., Shik M., Namintzer D., Vinayaka A.C., Ion A., Faigenboim A., Nasser A., Laish I. (2018). Identification of Synergistic Interaction Between Cannabis-Derived Compounds for Cytotoxic Activity in Colorectal Cancer Cell Lines and Colon Polyps That Induces Apoptosis-Related Cell Death and Distinct Gene Expression. Cannabis Cannabinoid Res..

[47] Santiago M., Sachdev S., Arnold J.C., Mcgregor I.S., Connor M. (2019). Absence of Entourage: Terpenoids Commonly Found inCannabis sativaDo Not Modulate the Functional Activity of Δ9-THC at Human CB1and CB2Receptors. Cannabis Cannabinoid Res..

[48] Finlay D.B., Sircombe K.J., Nimick M., Jones C., Glass M. (2020). Terpenoids From Cannabis Do Not Mediate an Entourage Effect by Acting at Cannabinoid Receptors. Front. Pharmacol..

[49] Heblinski M., Santiago M., Fletcher C., Stuart J., Connor M., McGregor I.S., Arnold J.C. (2020). Terpenoids Commonly Found in Cannabis sativa Do Not Modulate the Actions of Phytocannabinoids or Endocannabinoids on TRPA1 and TRPV1 Channels. Cannabis Cannabinoid Res..

[50] De Petrocellis L., Ligresti A., Moriello A.S., Allarà M., Bisogno T., Petrosino S., Stott C.G., Di Marzo V. (2011). Effects of cannabinoids and cannabinoid-enriched Cannabis extracts on TRP channels and endocannabinoid metabolic enzymes. Br. J. Pharmacol..

[51] Smart D., Gunthorpe M.J., Jerman J.C., Nasir S., Gray J., Muir A.I., Chambers J.K., Randall A., Davis J.B. (2000). The endogenous lipid anandamide is a full agonist at the human vanilloid receptor (hVR1). J. Cereb. Blood Flow Metab..

[52] De Petrocellis L., Di Marzo V. (2009). Non-CB1, Non-CB2 Receptors for Endocannabinoids, Plant Cannabinoids, and Synthetic Cannabimimetics: Focus on G-protein-coupled Receptors and Transient Receptor Potential Channels. J. Neuroimmune Pharmacol..

[53] Starkus J., Jansen C., Shimoda L.M.N., Stokes A.J., Small-Howard A.L., Turner H. (2019). Diverse TRPV1 responses to cannabinoids. Channels.

[54] Jansen C., Shimoda L.M.N., Kawakami J.K., Ang L., Bacani A.J., Baker J.D., Badowski C., Speck M., Strokes A.J., Small-Howard A.L. (2019). Myrcene and terpene regulation of TRPV1. Channels.

[55] Riera C.E., Menozzi-Smarrito C., Affolter M., Michlig S., Munari C., Robert F., Vogel H., Simon S.A., Le Coutre J. (2009). Compounds from Sichuan and Melegueta peppers activate, covalently and non-covalently, TRPA1 and TRPV1 channels. Br. J. Pharmacol..

[56] Galeotti N., Mannelli L.D.C., Mazzanti G., Bartolini A., Ghelardini C. (2001). Menthol: A natural analgesic compound. Neurosci. Lett..

[57] Liu B., Fan L., Balakrishna S., Sui A., Morris J.B., Jordt S.-E. (2013). TRPM8 is the principal mediator of menthol-induced analgesia of acute and inflammatory pain. Pain.

[58] Lowe H., Steele B., Bryant J., Toyang N., Ngwa W. (2021). Non-Cannabinoid Metabolites of *Cannabis sativa* L. with Therapeutic Potential. Plants.

[59] Cardinali A., Lattanzio V., Lattanzio V.M.T. (2006). Role of Phenolics in the Resistance Mechanisms of Plants against Fungal Pathogens and Insects. Res. Signpost.

[60] Manach C., Scalbert A., Morand C., Rémésy C., Jiménez L. (2004). Polyphenols: Food sources and bioavailability. Am. J. Clin. Nutr..

[61] Hart J.H. (1974). Inhibition of Wood-Rotting Fungi by Stilbenes and Other Polyphenols in Eucaly ptus sideroxylon. Phytopathology.

[62] El Gharras H. (2009). Polyphenols: Food sources, properties and applications—A review. Int. J. Food Sci. Technol..

[63] Carullo G., Cappello A.R., Frattaruolo L., Badolato M., Armentano B., Aiello F. (2017). Quercetin and derivatives: Useful tools in inflammation and pain management. Futur. Med. Chem..

[64] Tapas A., Sakarkar D., Kakde R. (2008). Flavonoids as Nutraceuticals: A Review. Trop. J. Pharm. Res..

[65] Leyva-López N., Gutierrez-Grijalva E.P., Ambriz-Perez D.L., Heredia J.B. (2016). Flavonoids as Cytokine Modulators: A Possible Therapy for Inflammation-Related Diseases. Int. J. Mol. Sci..

[66] Fürst R., Zündorf I. (2014). Plant-Derived Anti-Inflammatory Compounds: Hopes and Disappointments regarding the Translation of Preclinical Knowledge into Clinical Progress. Mediat. Inflamm..

[67] Hirpara K.V., Aggarwal P., Mukherjee A.J., Joshi N., Burman A.C. (2009). Quercetin and Its Derivatives: Synthesis, Pharmacological Uses with Special Emphasis on Anti-Tumor Properties and Prodrug with Enhanced Bio-Availability. Anti-Cancer Agents Med. Chem..

[68] D’Andrea G. (2015). Quercetin: A flavonol with multifaceted therapeutic applications?. Fitoterapia.

[69] Shrinivasan M., Skariyachan S., Aparna V., Kolte V.R. (2012). Homology modelling of CB1 receptor and selection of potential inhibitor against Obesity. Bioinformation.

[70] Refolo M.G., D’Alessandro R., Malerba N., Laezza C., Bifulco M., Messa C., Caruso M.G., Notarnicola M., Tutino V. (2015). Anti Proliferative and Pro Apoptotic Effects of Flavonoid Quercetin Are Mediated by CB1 Receptor in Human Colon Cancer Cell Lines. J. Cell. Physiol..

[71] Tutino V., DE Nunzio V., Tafaro A., Bianco G., Gigante I., Scavo M.P., D’Alessandro R., Refolo M.G., Messa C., Caruso M.G. (2018). Cannabinoid Receptor-1 Up-regulation in Azoxymethane (AOM)-treated Mice After Dietary Treatment with Quercetin. Anticancer. Res..

[72] Lin R., Piao M., Song Y., Liu C. (2020). Quercetin Suppresses AOM/DSS-Induced Colon Carcinogenesis through Its Anti-Inflammation Effects in Mice. J. Immunol. Res..

[73] Wang D., Wang H., Ning W., Backlund M.G., Dey S.K., Dubois R.N. (2008). Loss of Cannabinoid Receptor 1 Accelerates Intestinal Tumor Growth. Cancer Res..

[74] Sun A.Y., Wang Q., Simonyi A., Sun G.Y. (2010). Resveratrol as a Therapeutic Agent for Neurodegenerative Diseases. Mol. Neurobiol..

[75] Bournival J., Quessy P., Martinoli M.-G. (2009). Protective Effects of Resveratrol and Quercetin Against MPP+ -Induced Oxidative Stress Act by Modulating Markers of Apoptotic Death in Dopaminergic Neurons. Cell. Mol. Neurobiol..

[76] Bertelli A., Bertelli A.A., Gozzini A., Giovannini L. (1998). Plasma and Tissue Resveratrol Concentrations and Pharmacological Activity. Drugs Exp. Clin. Res..

[77] Walle T., Hsieh F., DeLegge M.H., Oatis J.E., Walle U.K. (2004). High absorption but very low bioavailability of oral resveratrol in humans. Drug Metab. Dispos..

[78] Chen M., Hou P., Zhou M., Ren Q., Wang X., Huang L., Hui S., Yi L., Mi M. (2019). Resveratrol attenuates high-fat diet-induced non-alcoholic steatohepatitis by maintaining gut barrier integrity and inhibiting gut inflammation through regulation of the endocannabinoid system. Clin. Nutr..

[79] Cani P.D., Plovier H., Van Hul M., Geurts L., Delzenne N.M., Druart C., Everard A. (2016). Endocannabinoids—At the crossroads between the gut microbiota and host metabolism. Nat. Rev. Endocrinol..

[80] Pertwee R.G., Howlett A.C., Abood M.E., Alexander S.P., Di Marzo V., Elphick M.R., Greasley P.J., Hansen H.S., Kunos G., Mackie K. (2010). International Union of Basic and Clinical Pharmacology. LXXIX. Cannabinoid Receptors and Their Ligands: Beyond CB_1_ and CB_2_. Pharmacol. Rev..

[81] Hassanzadeh P., Arbabi E., Atyabi F., Dinarvand R. (2016). The endocannabinoid system and NGF are involved in the mechanism of action of resveratrol: A multi-target nutraceutical with therapeutic potential in neuropsychiatric disorders. Psychopharmacology.

[82] Carta G., Poddighe L., Serra M.P., Boi M., Melis T., Lisai S., Murru E., Muredda L., Collu M., Banni S. (2018). Preventive Effects of Resveratrol on Endocannabinoid System and Synaptic Protein Modifications in Rat Cerebral Cortex Challenged by Bilateral Common Carotid Artery Occlusion and Reperfusion. Int. J. Mol. Sci..

[83] Oliveira C.D.C., e Castor M.G.M., e Castor C.G.M., Costa D.F., Ferreira R.C.M., Silva J., Navia-Pelaez J.M., Capettini L.D.S.A., Lemos V.S., Duarte I.D.G. (2019). Evidence for the involvement of opioid and cannabinoid systems in the peripheral antinociception mediated by resveratrol. Toxicol. Appl. Pharmacol..

[84] Hourani W., Alexander S.P.H. (2018). Cannabinoid ligands, receptors and enzymes: Pharmacological tools and therapeutic potential. Brain Neurosci. Adv..

[85] Hill M.N., Gorzalka B.B. (2005). Pharmacological enhancement of cannabinoid CB1 receptor activity elicits an antidepressant-like response in the rat forced swim test. Eur. Neuropsychopharmacol..

[86] Zanettini C., Panlilio L.V., Aliczki M., Goldberg S.R., Haller J., Yasar S. (2011). Effects of endocannabinoid system modulation on cognitive and emotional behavior. Front. Behav. Neurosci..

[87] Cebeci F., Şahin-Yeşilçubuk N. (2013). The matrix effect of blueberry, oat meal and milk on polyphenols, antioxidant activity and potential bioavailability. Int. J. Food Sci. Nutr..

[88] Ahmad H., Rauf K., Zada W., McCarthy M., Abbas G., Anwar F., Shah A.J. (2020). Kaempferol Facilitated Extinction Learning in Contextual Fear Conditioned Rats via Inhibition of Fatty-Acid Amide Hydrolase. Molecules.

[89] Haller J., Barna I., Barsvari B., Pelczer K.G., Yasar S., Panlilio L.V., Goldberg S. (2009). Interactions between environmental aversiveness and the anxiolytic effects of enhanced cannabinoid signaling by FAAH inhibition in rats. Psychopharmacology.

[90] Pamplona F. Letters RT-N, 2006 Undefined. WIN 55212-2 Impairs Contextual Fear Conditioning through the Activation of CB1 Cannabinoid Receptors. Elsevier. https://www.sciencedirect.com/science/article/pii/S030439400501390X?casa_token=Z3rlu2a_d_UAAAAA:vpWV0cYjbyvluJzrLoOki0IXgoIo2PpfnsQUWTE8_Q6EY5gN9cjPwPzL4MAqaLeCUq21NkcE.

[91] Thors L., Burston J., Alter B., McKinney M., Cravatt B., Ross R., Pertwee R., Th R.G., Wiley J., Fowler C. (2010). Biochanin A, a naturally occurring inhibitor of fatty acid amide hydrolase. Br. J. Pharmacol..

[92] Scholz, Williamson (2007). Interactions Affecting the Bioavailability of Dietary Polyphenols in vivo. Int. J. Vitam. Nutr. Res..

[93] Cocci P., Moruzzi M., Martinelli I., Maggi F., Di Bonaventura M.V.M., Cifani C., Mosconi G., Tayebati S.K., Damiano S., Lupidi G. (2021). Tart cherry (*Prunus cerasus* L.) dietary supplement modulates visceral adipose tissue CB1 mRNA levels along with other adipogenesis-related genes in rat models of diet-induced obesity. Eur. J. Nutr..

[94] Wojdyło A., Nowicka P., Laskowski P., Oszmiański J. (2014). Evaluation of Sour Cherry (Prunus cerasus L.) Fruits for Their Polyphenol Content, Antioxidant Properties, and Nutritional Components. J. Agric. Food Chem..

[95] Yılmaz F.M., Görgüç A., Karaaslan M., Vardin H., Bilek S.E., Uygun Ö., Bircan C. (2018). Sour Cherry By-products: Compositions, Functional Properties and Recovery Potentials—A Review. Crit. Rev. Food Sci. Nutr..

[96] Lee Y., Tharp W.G., Dixon A.E., Spaulding L., Trost S., Nair S., Permana P.A., Pratley R.E. (2009). Dysregulation of cannabinoid CB1 receptor expression in subcutaneous adipocytes of obese individuals. Anim. Cells Syst..

[97] Börner C., Bedini A., Höllt V., Kraus J. (2007). Analysis of Promoter Regions Regulating Basal and Interleukin-4-Inducible Expression of the Human CB1 Receptor Gene in T Lymphocytes. Mol. Pharmacol..

[98] Börner C., Höllt V., Sebald W., Kraus J. (2006). Transcriptional regulation of the cannabinoid receptor type 1 gene in T cells by cannabinoids. J. Leukoc. Biol..

[99] Russo G.L., Siani A., Fogliano V., Geleijnse J.M., Giacco R., Giampaoli S., Iacoviello L., Kromhout D., Lionetti L., Naska A. (2021). The Mediterranean diet from past to future: Key concepts from the second “Ancel Keys” International Seminar. Nutr. Metab. Cardiovasc. Dis..

[100] Bulló M., Casas R., Portillo M., Basora J., Estruch R., García-Arellano A., Lasa A., Juanola-Falgarona M., Arós F., Salas-Salvadó J. (2013). Dietary glycemic index/load and peripheral adipokines and inflammatory markers in elderly subjects at high cardiovascular risk. Nutr. Metab. Cardiovasc. Dis..

[101] Babio N., Toledo E., Estruch R., Ros E., Martínez-González M.A., Castañer O., Bulló M., Corella D., Arós F., Gómez-Gracia E. (2014). Mediterranean diets and metabolic syndrome status in the PREDIMED randomized trial. Can. Med Assoc. J..

[102] Toledo E., Salas-Salvadó J., Donat-Vargas C., Buil-Cosiales P., Estruch R., Ros E., Corella D., Fitó M., Hu F.B., Arós F. (2015). Mediterranean Diet and Invasive Breast Cancer Risk Among Women at High Cardiovascular Risk in the PREDIMED Trial: A Randomized Clinical Trial. JAMA Intern. Med..

[103] de Pablos R.M., Espinosa-Oliva A.M., Hornedo-Ortega R., Cano M., Arguelles S. (2019). Hydroxytyrosol protects from aging process via AMPK and autophagy; a review of its effects on cancer, metabolic syndrome, osteoporosis, immune-mediated and neurodegenerative diseases. Pharmacol. Res..

[104] Castro-Barquero S., Lamuela-Raventós R.M., Doménech M., Estruch R. (2018). Relationship between Mediterranean Dietary Polyphenol Intake and Obesity. Nutrients.

[105] Dai J., Mumper R.J. (2010). Plant Phenolics: Extraction, Analysis and Their Antioxidant and Anticancer Properties. Molecules.

[106] Hornedo-Ortega R., Cerezo A.B., De Pablos R.M., Krisa S., Richard T., García-Parrilla M.C., Troncoso A.M. (2018). Phenolic Compounds Characteristic of the Mediterranean Diet in Mitigating Microglia-Mediated Neuroinflammation. Front. Cell. Neurosci..

[107] Notarnicola M., Pisanti S., Tutino V., Bocale D., Rotelli M.T., Gentile A., Memeo V., Bifulco M., Perri E., Caruso M.G. (2010). Effects of olive oil polyphenols on fatty acid synthase gene expression and activity in human colorectal cancer cells. Genes Nutr..

[108] Francesco A., Di Falconi A., Di Germanio C., Di Bonaventura M.V.M., Costa A., Caramuta S., Del Carlo M., Compagnone D., Dainese E., Cifani C. CDG-TJ of Nutritional, 2015 Undefined. Extravirgin Olive Oil Up-Regulates CB1 Tumor Suppressor Gene in Human Colon Cancer Cells and in Rat Colon via Epigenetic Mechanisms. Elsevier. https://www.sciencedirect.com/science/article/pii/S0955286314002411?casa_token=bBNECjqYZBsAAAAA:w4ZuUKtZ6KQT3FbQZuW2y2ZnTTMRZ_e9RtbBKnzg46OLsjoF3N2eo3Z51Jo_Q3TEbrV4K3_E.

[109] Tutino V., Orlando A., Russo F., Notarnicola M. (2015). Hydroxytyrosol Inhibits Cannabinoid CB1 Receptor Gene Expression in 3T3-L1 Preadipocyte Cell Line. J. Cell. Physiol..

[110] You T., Disanzo B.L., Wang X., Yang R., Gong D. (2011). Adipose tissue endocannabinoid system gene expression: Depot differences and effects of diet and exercise. Lipids Heal. Dis..

[111] Roche R., Hoareau L., Bes-Houtmann S., Gonthier M.-P., Laborde C., Baron J.-F., Haffaf Y., Cesari M., Festy F. (2006). Presence of the cannabinoid receptors, CB1 and CB2, in human omental and subcutaneous adipocytes. Histochem. Cell Biol..

[112] Notarnicola M., Tutino V., Tafaro A., Bianco G., Guglielmi E., Caruso M.G. (2016). Dietary olive oil induces cannabinoid CB2 receptor expression in adipose tissue of ApcMin/+ transgenic mice. Nutr. Health Aging.

[113] Hervera A., Negrete R., Leánez S., Martín-Campos J., Pol O. The Role of Nitric Oxide in the Local Antiallodynic and Antihyperalgesic Effects and Expression of δ-Opioid and Cannabinoid-2 Receptors during Neuropathic Pain in Mice. ASPET. https://jpet.aspetjournals.org/content/334/3/887.short.

[114] Sakkas H., Bozidis P., Touzios C., Kolios D., Athanasiou G., Athanasopoulou E., Gerou I., Gartzonika C. (2020). Nutritional Status and the Influence of the Vegan Diet on the Gut Microbiota and Human Health. Medicina.

[115] Paoli A., Mancin L., Bianco A., Thomas E., Mota J.F., Piccini F. (2019). Ketogenic Diet and Microbiota: Friends or Enemies?. Genes.

[116] Zmora N., Suez J., Elinav E. (2018). You are what you eat: Diet, health and the gut microbiota. Nat. Rev. Gastroenterol. Hepatol..

[117] Nagpal R., Shively C.A., Register T.C., Craft S., Yadav H. (2019). Gut microbiome-mediterranean diet interactions in improving host health [version 1; peer review: 1 approved]. F1000Research.

[118] Tosti V., Bertozzi B., Fontana L. (2018). Health Benefits of the Mediterranean Diet: Metabolic and Molecular Mechanisms. J. Gerontol. A Biol. Sci. Med. Sci..

[119] Cani P.D. (2012). Crosstalk between the gut microbiota and the endocannabinoid system: Impact on the gut barrier function and the adipose tissue. Clin. Microbiol. Infect..

[120] Mehrpouya-Bahrami P., Chitrala K.N., Ganewatta M.S., Tang C., Murphy E.A., Enos R.T., Velazquez K.T., McCellan J., Nagarkatti M., Nagarkatti P. (2017). Blockade of CB1 cannabinoid receptor alters gut microbiota and attenuates inflammation and diet-induced obesity. Sci. Rep..

[121] Everard A., Belzer C., Geurts L., Ouwerkerk J.P., Druart C., Bindels L.B., Guiot Y., Derrien M., Muccioli G.G., Delzenne N.M. (2013). Cross-talk between *Akkermansia muciniphila* and intestinal epithelium controls diet-induced obesity. Proc. Natl. Acad. Sci. USA.

[122] Cani P., Possemiers S., Wiele T., Van de Gut Y.G. 2009 undefined. Changes in Gut Microbiota Control Inflammation in Obese Mice through a Mechanism Involving GLP-2-Driven Improvement of gut Permeability. gut.bmj.com. https://gut.bmj.com/content/58/8/1091.short.

[123] Kameyama K., Itoh K. (2014). Intestinal Colonization by a Lachnospiraceae Bacterium Contributes to the Development of Diabetes in Obese Mice. Microbes Environ..

[124] Kaakoush N.O. (2015). Insights into the Role of Erysipelotrichaceae in the Human Host. Front. Cell. Infect. Microbiol..

[125] Iannotti F., Piscitelli F., Martella A., Mazzarella E., Allarà M., Palmieri V., Parrella C., Capasso R., Di Marzo V. (2013). Analysis of the “endocannabinoidome” in peripheral tissues of obese Zucker rats. Prostaglandins Leukot. Essent. Fat. Acids.

[126] Syed S.K., Bui H.H., Beavers L.S., Farb T.B., Ficorilli J., Chesterfield A.K., Kuo M.-S., Bokvist K., Barrett D.G., Efanov A. (2012). Regulation of GPR119 receptor activity with endocannabinoid-like lipids. Am. J. Physiol. Metab..

[127] Muccioli G.G., Naslain D., Bäckhed F., Reigstad C.S., Lambert D.M., Delzenne N.M., Cani P.D. (2010). The endocannabinoid system links gut microbiota to adipogenesis. Mol. Syst. Biol..

[128] Geurts L., Everard A., Van Hul M., Essaghir A., Duparc T., Matamoros S., Plovier H., Castel J., Denis R., Bergiers M. (2015). Adipose tissue NAPE-PLD controls fat mass development by altering the browning process and gut microbiota. Nat. Commun..

[129] Alhouayek M., Lambert D.M., Delzenne N.M., Cani P.D., Muccioli G.G. (2011). Increasing endogenous 2-arachidonoylglycerol levels counteracts colitis and related systemic inflammation. FASEB J..

[130] Murataeva N., Dhopeshwarkar A., Yin D., Mitjavila J., Bradshaw H., Straiker A., Mackie K. (2016). Where’s my entourage? The curious case of 2-oleoylglycerol, 2-linolenoylglycerol, and 2-palmitoylglycerol. Pharmacol. Res..

[131] Kang C., Wang B., Kaliannan K., Wang X., Lang H., Hui S., Huang L., Zhang Y., Zhou M., Chen M. (2017). Gut Microbiota Mediates the Protective Effects of Dietary Capsaicin against Chronic Low-Grade Inflammation and Associated Obesity Induced by High-Fat Diet. mBio.

[132] Cantu-Jungles T.M., Rasmussen H.E., Hamaker B.R. (2019). Potential of Prebiotic Butyrogenic Fibers in Parkinson’s Disease. Front. Neurol..

[133] Scott K.P., Martin J.C., Duncan S., Flint H.J. (2013). Prebiotic stimulation of human colonic butyrate-producing bacteria and bifidobacteria, in vitro. FEMS Microbiol. Ecol..

[134] La Rosa S.L., Kachrimanidou V., Buffetto F., Pope P.B., Pudlo N.A., Martens E.C., Rastall R.A., Gibson G.R., Westereng B. (2019). Wood-Derived Dietary Fibers Promote Beneficial Human Gut Microbiota. mSphere.

[135] Markey L., Hooper A., Melon L.C., Baglot S., Hill M.N., Maguire J., Kumamoto C.A. (2020). Colonization with the commensal fungus Candida albicans perturbs the gut-brain axis through dysregulation of endocannabinoid signaling. Psychoneuroendocrinology.

[136] Lacroix S., Pechereau F., Leblanc N., Boubertakh B., Houde A., Martin C., Flamand N., Silvestri C., Raymond F., Di Marzo V. (2019). Rapid and Concomitant Gut Microbiota and Endocannabinoidome Response to Diet-Induced Obesity in Mice. mSystems.

[137] Barutta F., Piscitelli F., Pinach S., Bruno G., Gambino R., Rastaldi M.P., Salvidio G., Di Marzo V., Perin P.C., Gruden G. (2011). Protective Role of Cannabinoid Receptor Type 2 in a Mouse Model of Diabetic Nephropathy. Diabetes.

[138] Barutta F., Grimaldi S., Franco I., Bellini S., Gambino R., Pinach S., Corbelli A., Bruno G., Rastaldi M.P., Aveta T. (2014). Deficiency of cannabinoid receptor of type 2 worsens renal functional and structural abnormalities in streptozotocin-induced diabetic mice. Kidney Int..

